# Personality traits and anxiety in a Tibetan cultural context: incremental contributions of neuroticism and extraversion

**DOI:** 10.3389/fpsyg.2026.1906142

**Published:** 2026-08-28

**Authors:** Jialu Ye, Jie Zhang, Jiafeng Li, Yushu Tang, Qingrong Liu, Lijun Jiang

**Affiliations:** 1 Mental Health Center, West China Hospital of Sichuan University, Chengdu, China; 2 National Center for Mental Disorders, West China Hospital, Sichuan University, Chengdu, China; 3 General Practice Ward/International Medical Center Ward, General Practice Medical Center, West China Hospital, Sichuan University, Chengdu, China; 4 West China School of Nursing, Sichuan University, Chengdu, China; 5 Monash University, Melbourne, VIC, Australia; 6 Sichuan Provincial Corps Hospital, Chinese People's Armed Police Forces, Leshan, China

**Keywords:** anxiety symptoms, cross-cultural psychology, extraversion, neuroticism, Tibetan population

## Abstract

**Background:**

Personality traits are well-established correlates of anxiety symptoms, yet whether these associations generalize across culturally distinct populations remains insufficiently understood. This study examined the incremental explanatory value of neuroticism and extraversion for anxiety symptoms in a Tibetan sociocultural context after accounting for sociodemographic, health-related, and social-contextual factors.

**Methods:**

A cross-sectional survey was conducted among 583 community-dwelling Tibetan adults recruited through convenience sampling in western China. Participants completed the Tibetan adaptations of the Brief Big Five Personality Scale and the Generalized Anxiety Disorder-7 (GAD-7), together with measures of sociodemographic characteristics, mental health history, perceived social support, and religious affiliation. Given the absence of published psychometric validation of the Tibetan adaptation, internal consistency was assessed and an exploratory factor analysis (EFA) was conducted to evaluate the reliability and underlying factor structure of the personality measure. Hierarchical multiple regression and exploratory moderation analyses examined the incremental explanatory value of personality traits and the potential moderating effects of perceived social support and religious affiliation.

**Results:**

Neuroticism and extraversion explained an additional 34.3% of the variance in anxiety symptoms beyond sociodemographic and health-related characteristics, increasing the total explained variance from 5.0 to 39.3%. Both traits were positively associated with anxiety symptoms. Perceived social support and religious affiliation contributed negligible additional explanatory value and did not moderate the association between neuroticism and anxiety symptoms. EFA supported a theoretically informed two-factor solution despite substantial overlap between neuroticism and extraversion.

**Conclusion:**

Personality traits provided substantial incremental explanatory value for anxiety symptoms in this Tibetan sample. Neuroticism emerged as a robust correlate of anxiety, whereas extraversion was also positively associated with anxiety symptoms, a pattern differing from findings commonly reported in Western populations. These findings highlight the importance of interpreting personality–anxiety associations within their sociocultural and psychometric context and underscore the need for culturally validated personality measures when extending personality research to understudied populations.

## 1 Introduction

Anxiety symptoms are among the most prevalent mental health concerns worldwide and are associated with substantial impairments in mental health, daily functioning, and quality of life. They are also linked to increased psychological comorbidity and healthcare utilization (Bandelow and Michaelis, 2015; Baxter et al., 2013). Given their considerable individual and societal burden, identifying factors that contribute to individual differences in anxiety symptoms remains a major objective of mental health research.

Personality traits have long been recognized as important correlates of emotional functioning and vulnerability to anxiety. Within the Five-Factor Model (FFM), neuroticism reflects emotional instability, heightened stress sensitivity, and a tendency toward negative affect, whereas extraversion is characterized by sociability, positive emotionality, and engagement with the external environment. A substantial body of evidence indicates that higher neuroticism is associated with greater anxiety symptoms (Kotov et al., 2010; Jeronimus et al., 2016), whereas extraversion is generally regarded as a protective characteristic associated with lower levels of anxiety and better psychological adjustment (Malouff et al., 2005). Nevertheless, most evidence regarding these associations has been derived from Western populations, and the extent to which these relationships generalize across different cultural contexts remains insufficiently understood.

Personality–anxiety associations may vary across sociocultural settings (Markus and Kitayama, 1991). Tibetan communities represent an important yet understudied sociocultural context characterized by strong Buddhist traditions, collectivistic social organization, close-knit interpersonal networks, and culturally embedded norms emphasizing social harmony, interpersonal obligation, and religious practice (Samuel, 1993; Childs, 2004; Zhou et al., 2025). These characteristics may shape emotional expression, coping strategies, and the behavioral manifestations of personality traits (Cheung et al., 2011), potentially giving rise to personality–anxiety associations that differ from those observed in predominantly Western populations (McCrae and Terracciano, 2005). Accordingly, findings derived from Western populations may not readily generalize to Tibetan communities. Furthermore, to our knowledge, whether widely used personality measures demonstrate comparable psychometric structures and construct validity in Tibetan populations has not been investigated.

In addition to personality traits, broader social-contextual factors may also contribute to anxiety symptoms. Social support has consistently been conceptualized as a protective factor that mitigates psychological distress by providing emotional, informational, and practical resources, Cohen and Wills, 1985 consistent with the stress-buffering hypothesis. Religious affiliation and religious involvement may likewise promote psychological well-being through meaning-making, emotion regulation, adaptive coping, and social integration within religious communities (Koenig et al., 2020; Koenig, 2018). However, although both social support and religiosity have been associated with anxiety and psychological well-being, previous findings remain inconsistent regarding whether these contextual factors explain anxiety beyond the influence of personality traits or buffer the association between neuroticism and anxiety symptoms. Clarifying the relative and interactive contributions of personality traits and social-contextual factors may therefore contribute to a more culturally informed understanding of anxiety.

Despite extensive evidence linking personality traits to anxiety symptoms, important questions remain regarding the extent to which these associations generalize across sociocultural settings and whether personality traits retain their explanatory value after broader social-contextual factors are taken into account. Tibetan communities provide a particularly informative context in which to address these questions because both personality expression and social participation may be shaped by distinctive cultural and religious traditions. Examining personality and contextual variables within the same analytical framework therefore provides an opportunity to evaluate whether personality contributes unique explanatory value beyond broader demographic and social influences while also informing the cross-cultural generalizability of personality–anxiety associations.

The present study examined the incremental contribution of personality traits to anxiety symptoms in a Tibetan sociocultural context. Specifically, we investigated whether neuroticism and extraversion explained additional variance in GAD-7 total scores beyond sociodemographic and health-related characteristics and whether social support and religious affiliation provided additional explanatory value after personality traits had been taken into account. Exploratory moderation analyses were conducted to examine whether social support and religious affiliation moderated the association between neuroticism and anxiety symptoms. Given the absence of published psychometric validation evidence for the Tibetan adaptation of the Brief Big Five Personality Scale, exploratory factor analysis (EFA) was also performed to examine the underlying structure of the personality items in the present sample. We hypothesized that neuroticism would be positively associated with anxiety symptoms and would remain a significant predictor after adjustment for sociodemographic and health-related characteristics. Because evidence from Tibetan populations remains limited and the psychological implications of extraversion may vary across sociocultural settings, no directional hypothesis was specified regarding the association between extraversion and anxiety symptoms.

## 2 Methods

### 2.1 Participants and procedure

This cross-sectional study employed a convenience sampling design. Participants were recruited through paper-based surveys administered in Tibetan communities located in high-altitude regions of western China. Eligible participants were community-dwelling Tibetan adults aged 18 years or older whose primary language was Tibetan and who were able to complete the questionnaire independently.

A total of 583 valid questionnaires were included in the final analyses. The sample comprised 46.8% men, 44.4% women, and 8.7% of participants who preferred not to disclose their sex. Age was categorized into five predefined groups, with the largest proportion of participants aged 18–29 years. Additional sociodemographic and health-related information, including educational attainment, occupation, household income, mental health history, perceived social support, and religious affiliation, was obtained through self-report.

Questionnaires were administered anonymously by trained research personnel using standardized procedures. Before participation, all participants were informed of the study objectives, the voluntary nature of participation, confidentiality safeguards, and their right to withdraw at any time. Clarification was provided when necessary, although no assistance was given in answering questionnaire items. No personally identifiable information was collected. Questionnaires were screened for completeness before data entry, and those with substantial missing data were excluded.

### 2.2 Measures

#### 2.2.1 Sociodemographic and health-related characteristics

Participants reported sociodemographic and health-related characteristics, including age, sex, educational attainment, occupation, household income, mental health history, perceived social support, and religious affiliation. Mental health history was assessed using a single self-reported dichotomous item as a broad indicator of previous mental health problems rather than a clinically verified diagnosis. Perceived social support was assessed using a single self-reported categorical item reflecting participants’ overall perception of available social support. Religious affiliation was assessed using a binary self-report item (yes/no).

#### 2.2.2 Brief Big Five Personality Scale

Personality traits were assessed using the Tibetan adaptation of the Brief Big Five Personality Scale (Wang et al., 2011). Because neuroticism and extraversion have consistently demonstrated the strongest associations with anxiety symptoms in previous research, the present study focused on these two dimensions. To our knowledge, no published psychometric validation study of the Tibetan adaptation is currently available. The Tibetan version used in this study was developed through translation, back-translation, expert review, and cultural adaptation. Internal consistency of the neuroticism and extraversion subscales was evaluated using Cronbach’s *α* and McDonald’s *ω* total. Detailed psychometric properties are presented in the Results section and Supplementary Tables S1, S2.

#### 2.2.3 Generalized anxiety symptoms

Generalized anxiety symptoms were assessed using the Generalized Anxiety Disorder-7 (GAD-7) (Spitzer et al., 2006), a widely used self-report measure developed by Spitzer et al. to assess the severity of generalized anxiety symptoms during the preceding two weeks. The scale comprises seven items assessing core symptoms of generalized anxiety, including excessive worry, difficulty controlling worry, restlessness, irritability, and difficulty relaxing.

Each item is rated on a four-point scale ranging from 0 (“not at all”) to 3 (“nearly every day”), yielding total scores ranging from 0 to 21, with higher scores indicating greater anxiety severity.

The Tibetan version of the GAD-7 was used in the present study. The instrument underwent translation, back-translation, and cultural adaptation before administration. In the present sample, the GAD-7 demonstrated acceptable internal consistency (Cronbach’s *α* = 0.70).

Additional instrument characteristics are provided in Supplementary Table S1, and internal consistency indices are reported in Supplementary Table S2.

### 2.3 Statistical analysis

All statistical analyses were performed using IBM SPSS Statistics version 26, except McDonald’s *ω* total, which was estimated separately for the neuroticism and extraversion subscales using one-factor minimum residual models based on Pearson correlations in the psych package (version 2.6.5) in R (version 4.5.1). Descriptive statistics were computed for all study variables. Categorical variables are presented as frequencies and percentages, whereas continuous variables are presented as means and standard deviations (SDs). Pearson correlation coefficients were calculated to examine bivariate associations between GAD-7 total scores and all variables included in Model 1, as well as neuroticism and extraversion.

Covariates were selected *a priori* based on previous evidence indicating potential associations with anxiety symptoms. The order of model entry reflected the primary objective of evaluating the incremental explanatory value of personality traits beyond variables that are routinely considered in epidemiological studies of anxiety. Hierarchical multiple regression analyses were conducted to examine the incremental explanatory value of personality traits for GAD-7 total scores beyond sociodemographic and health-related characteristics. Model 1 included age, sex, educational attainment, occupation, household income, and mental health history. Model 2 additionally included neuroticism and extraversion to evaluate the incremental explanatory value of personality traits. Model 3 further included perceived social support and religious affiliation to examine whether these social-contextual factors explained additional variance beyond sociodemographic, health-related, and personality variables.

To evaluate the robustness of the regression models, sensitivity analyses were conducted by replacing the numerically coded sex and occupation variables with dummy-coded categorical variables. Male participants and agricultural/pastoral workers served as the reference groups. Detailed results of the sensitivity analyses are reported in Supplementary Table S6.

Standardized regression coefficients (*β*), coefficients of determination (R²), changes in explained variance (ΔR²), F statistics, and F-change statistics (ΔF) are reported. Multicollinearity was assessed using variance inflation factors (VIFs). Regression assumptions were further evaluated using normal probability plots of standardized residuals and scatterplots of standardized residuals against standardized predicted values. These diagnostic plots are presented in Supplementary Figures S1, S2.

Exploratory moderation analyses were conducted to examine whether perceived social support or religious affiliation moderated the association between neuroticism and anxiety symptoms. Neuroticism was mean-centered before interaction terms were created. The Neuroticism × Social Support and Neuroticism × Religion interaction terms were entered separately after the inclusion of covariates, personality traits, perceived social support, and religious affiliation. Detailed moderation results are reported in Supplementary Table S5.

Exploratory factor analysis (EFA) was conducted to examine the underlying structure of the personality items. Factorability was assessed using the Kaiser–Meyer–Olkin (KMO) measure and Bartlett’s test of sphericity. Principal axis factoring with Promax rotation was performed because correlations among latent personality dimensions were expected. Factor retention was initially evaluated using the Kaiser criterion (eigenvalues > 1) together with visual inspection of the scree plot. Although the Kaiser criterion suggested a four-factor solution, the scree plot indicated a clear inflection after the second factor. Accordingly, a theoretically informed two-factor solution representing neuroticism and extraversion was extracted and interpreted. Detailed EFA results are reported in Appendix F.

### 2.4 Ethical considerations

This study was approved by the Biomedical Research Ethics Committee of West China Hospital, Sichuan University (Approval No. 2026–011). Written informed consent was obtained from all participants before participation. All study procedures were conducted in accordance with the ethical principles of the Declaration of Helsinki.

## 3 Results

### 3.1 Participant characteristics

A total of 583 participants were included in the final analyses. Their sociodemographic and health-related characteristics are summarized in Table 1.

**Table 1:** Sociodemographic and health-related characteristics of the participants.

Variable	*n* (%)
Age group, *n* (%)	
18–29 years	277 (47.5)
30–39 years	156 (26.8)
40–49 years	97 (16.6)
50–59 years	32 (5.5)
60 years or above	21 (3.6)
Sex, *n* (%)	
Male	273 (46.8)
Female	259 (44.4)
Prefer not to say	51 (8.7)
Education, *n* (%)	
Primary school or below	216 (37.0)
Junior high school	131 (22.5)
High school/vocational school	147 (25.2)
Bachelor’s degree/college	64 (11.0)
Master’s degree or above	25 (4.3)
Occupation, *n* (%)	
Agricultural/pastoral worker	251 (43.1)
Manual/service worker	107 (18.4)
Administrative/professional/technical staff	109 (18.7)
Self-employed/freelancer	56 (9.6)
Student	23 (3.9)
Unemployed/retired	37 (6.3)
Income, *n* (%)	
Low-income	261 (44.8)
Lower-middle-income	184 (31.6)
Upper-middle-income	104 (17.8)
High-income	34 (5.8)
Mental health history, *n* (%)	
Yes	256 (43.9)
No	327 (56.1)
Religion, *n* (%)	
Yes	331 (56.8)
No	252 (43.2)
Social support, *n* (%)	
Low social support	319 (54.7)
Moderate social support	216 (37.0)
High social support	48 (8.2)

Percentages may not total 100 due to rounding.

The sample was predominantly young, with nearly half of the participants (47.5%) aged 18–29 years. More than half reported a religious affiliation (56.8%), and over half reported low perceived social support (54.7%).

### 3.2 Descriptive statistics and correlations among study variables

Descriptive statistics, internal consistency coefficients, and bivariate correlations with GAD-7 total scores are reported in Table 2. Pearson correlation coefficients with GAD-7 total score are presented in this table. The complete correlation matrix is provided in Supplementary Table S3.

**Table 2:** Descriptive statistics and bivariate correlations with GAD-7 total score.

Variable	Mean (SD)	α	r with GAD-7
Age	——	——	0.074
Sex	——	——	0.106*
Education	——	——	0.042
Occupation	——	——	0.006
Household income	——	——	0.193***
Mental health history	——	——	0.061
Neuroticism	2.93 (0.97)	0.67	0.60***
Extraversion	2.98 (0.96)	0.69	0.58***
GAD-7 total score	7.58 (4.52)	0.70	——

Pearson correlation coefficients with GAD-7 total score are reported. Means (SDs) are presented only for continuous variables. *p < 0.05, **p < 0.01, ***p < 0.001.

Internal consistency was modest for both personality subscales. Cronbach’s *α* and McDonald’s *ω* total were 0.67 and 0.67, respectively, for neuroticism, and 0.69 and 0.70, respectively, for extraversion (Supplementary Table S2).

Household income was positively correlated with GAD-7 total scores (r = 0.19, *p* < 0.001), whereas age, educational attainment, occupation, and mental health history were not significantly correlated with GAD-7 total scores. Sex showed a weak positive correlation with GAD-7 total scores (r = 0.11, *p* = 0.011). Both neuroticism (r = 0.60, *p* < 0.001) and extraversion (r = 0.58, *p* < 0.001) were positively associated with GAD-7 total scores. Neuroticism and extraversion were also strongly correlated with one another (r = 0.78, *p* < 0.001).

### 3.3 Exploratory factor analysis of personality items

Exploratory factor analysis (EFA) was conducted to examine the underlying structure of the personality items. The Kaiser–Meyer–Olkin (KMO) measure was 0.832, and Bartlett’s test of sphericity was significant (χ^2^ = 2083.37, *p* < 0.001), indicating that the correlation matrix was suitable for factor analysis.

**Figure 1:**
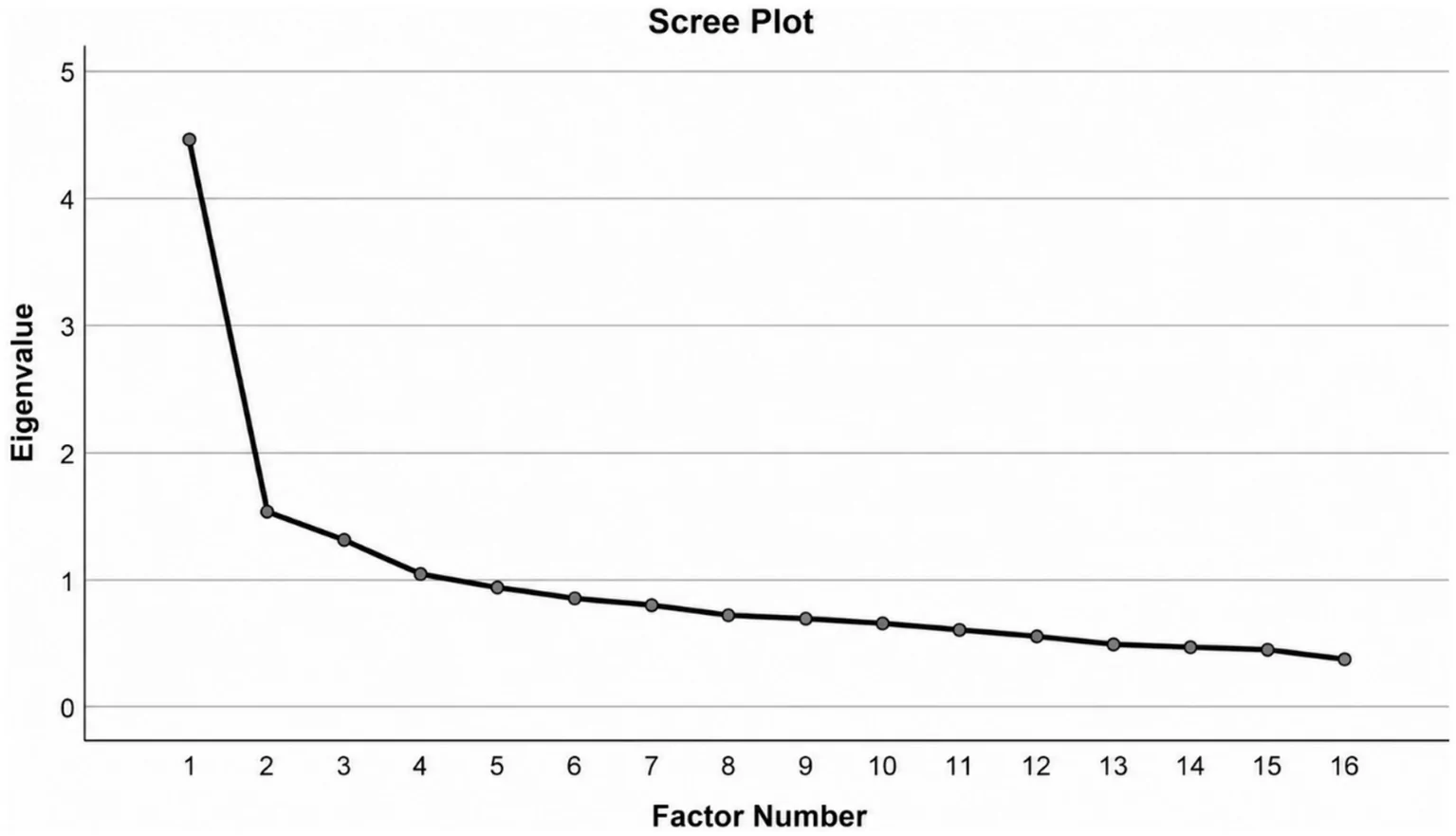
Scree plot of the exploratory factor analysis of the Brief Big Five Personality Scale items.

Initial factor extraction based on the Kaiser criterion (eigenvalues > 1) yielded a four-factor solution accounting for 52.33% of the total variance. However, inspection of the rotated factor pattern indicated that neuroticism- and extraversion-related items were distributed across multiple factors, and the fourth factor was defined by only two items, limiting its conceptual interpretability (Supplementary Table S9). By contrast, inspection of the scree plot (Figure 1) revealed a clear inflection after the second factor, supporting a more parsimonious solution. Accordingly, given the study’s *a priori* focus on neuroticism and extraversion, a theoretically informed two-factor solution was retained for interpretation.

The two retained factors accounted for 37.53% of the total variance. The rotated factor pattern revealed substantial overlap between neuroticism- and extraversion-related items, with several items loading on factors that did not fully correspond to their theoretical assignments. The two extracted factors were moderately correlated (r = 0.536), indicating considerable shared variance between the underlying dimensions. Detailed EFA results, including explained variance, rotated factor loadings, and factor correlations, are reported in Appendix F (Supplementary Tables S7–S10).

### 3.4 Hierarchical regression analyses predicting anxiety symptoms

Results of the hierarchical multiple regression analyses are presented in Table 3.

**Table 3:** Hierarchical multiple regression analyses predicting GAD-7 total scores.

Predictor	Model 1	Model 2	Model 3
Age	0.022	−0.012	−0.013
Sex	0.055	−0.046	−0.039
Education	−0.076	−0.024	−0.024
Occupation	−0.053	0.002	0.001
Household income	0.227***	0.065	0.068
Mental health history	0.036	0.02	0.028
Neuroticism	——	0.368***	0.368***
Extraversion	——	0.287***	0.289***
Social support	——	——	−0.020
Religious affiliation	——	——	−0.020
R^2^	0.050	0.393	0.394
ΔR^2^	0.050	0.343	0.001
F	F (6, 576) = 5.04***	F (8, 574) = 46.44 ***	F (10, 572) = 37.12***
ΔF	——	ΔF (2, 574) = 162.16***	ΔF (2, 572) = 0.29
VIF range	1.04–1.75	1.04–2.64	1.21–2.64

Standardized regression coefficients (β) are reported. Model 1 included sociodemographic and health-related covariates. Model 2 additionally included neuroticism and extraversion. Model 3 additionally included social support and religious affiliation. VIF = variance inflation factor. *p < 0.05, **p < 0.01, ***p < 0.001.

In Model 1, sociodemographic and health-related characteristics explained 5.0% of the variance in GAD-7 total scores (R^2^ = 0.050, *F* (6, 576) = 5.04, *p* < 0.001). Household income was the only significant predictor (*β* = 0.227, *p* < 0.001).

The addition of neuroticism and extraversion in Model 2 significantly improved model fit, explaining an additional 34.3% of the variance in GAD-7 total scores (ΔR^2^ = 0.343, ΔF (2, 574) = 162.16, *p* < 0.001). The total explained variance increased to 39.3% (R^2^ = 0.393, *F* (8, 574) = 46.44, *p* < 0.001). Both neuroticism (*β* = 0.368, *p* < 0.001) and extraversion (*β* = 0.287, *p* < 0.001) were significant positive predictors of GAD-7 total scores.

In Model 3, perceived social support and religious affiliation were entered as social-contextual variables. Their inclusion did not significantly improve model fit (ΔR^2^ = 0.001, ΔF (2, 572) = 0.29, *p* = 0.750), increasing the total explained variance only marginally (R^2^ = 0.394, *F* (10, 572) = 37.12, *p* < 0.001). Neither perceived social support (*β* = −0.020, *p* = 0.585) nor religious affiliation (*β* = −0.020, *p* = 0.585) was significantly associated with GAD-7 total scores after adjustment for sociodemographic, health-related, and personality variables.

Full regression coefficients are reported in Supplementary Table S4.

Multicollinearity diagnostics indicated no evidence of problematic collinearity, with VIF values ranging from 1.04 to 2.64 across all models. Although neuroticism and extraversion were strongly correlated at the bivariate level, all VIF values remained well below commonly recommended thresholds. Visual inspection of the normal P–P plot and the scatterplot of standardized residuals against standardized predicted values (Supplementary Figures S1, S2) revealed no substantial violations of regression assumptions.

Sensitivity analyses using dummy-coded representations of sex and occupation yielded substantively similar results (Supplementary Table S6). Neuroticism and extraversion remained significant positive predictors of GAD-7 total scores, whereas perceived social support and religious affiliation remained non-significant. The inclusion of social-contextual variables explained negligible additional variance (ΔR^2^ < 0.001, ΔF = 0.11, *p* = 0.895), supporting the robustness of the primary findings.

### 3.5 Exploratory moderation analyses

Exploratory moderation analyses were conducted to examine whether perceived social support or religious affiliation moderated the association between neuroticism and anxiety symptoms.

Neither the Neuroticism × Social Support interaction (*β* = −0.009, *p* = 0.918) nor the Neuroticism × Religious Affiliation interaction (*β* = 0.162, *p* = 0.120) reached statistical significance. The interaction terms explained negligible additional variance in GAD-7 total scores, providing no evidence that either perceived social support or religious affiliation moderated the association between neuroticism and anxiety symptoms in the present sample. Detailed regression coefficients are reported in Supplementary Table S5.

## 4 Discussion

### 4.1 Main findings

The present study examined the incremental explanatory value of personality traits for anxiety symptoms in a Tibetan sociocultural context. Four principal findings emerged. First, neuroticism remained a robust positive predictor of anxiety symptoms after adjustment for sociodemographic and health-related characteristics. Second, extraversion was unexpectedly positively associated with anxiety symptoms, a pattern that contrasts with findings commonly reported in Western populations. Third, perceived social support and religious affiliation provided negligible additional explanatory value beyond personality traits and did not moderate the association between neuroticism and anxiety symptoms. Finally, exploratory factor analysis revealed substantial overlap between neuroticism and extraversion, suggesting that the organization of these personality dimensions in the present sample differed from the canonical structure typically reported in Western populations. Collectively, these findings suggest that personality–anxiety associations may be more context-dependent than is commonly assumed and underscore the importance of considering both sociocultural influences and measurement issues when extending personality research to understudied populations.

### 4.2 Neuroticism as a robust predictor of anxiety symptoms

Neuroticism emerged as one of the strongest predictors of anxiety symptoms in the present study. This finding is consistent with extensive evidence demonstrating that neuroticism is closely associated with vulnerability to anxiety-related distress (Pawlak et al., 2025; Ormel et al., 2013). Moreover, the association remained robust after adjustment for sociodemographic, health-related, and broader social-contextual characteristics, suggesting that neuroticism represents a relatively stable dispositional vulnerability to anxiety symptoms across diverse cultural settings.

The present findings are also consistent with contemporary models of emotional vulnerability, which propose that individuals with higher levels of neuroticism are more likely to experience persistent negative affect, heightened stress sensitivity, and maladaptive emotional responses to everyday stressors (Barlow et al., 2014; Clark and Watson, 1991). Taken together, these findings reinforce previous evidence that neuroticism represents a broadly applicable vulnerability factor for anxiety while extending this evidence to an understudied Tibetan population.

### 4.3 Positive association between extraversion and anxiety symptoms

A noteworthy finding of the present study was the positive association between extraversion and anxiety symptoms. This pattern contrasts with evidence from many Western populations, in which extraversion is generally regarded as a protective personality characteristic associated with positive affect, social confidence, and lower levels of psychological distress (Steel et al., 2008; Anglim et al., 2020). In the present sample, however, extraversion remained a significant positive predictor of anxiety symptoms after adjustment for sociodemographic, health-related, personality, and social-contextual characteristics.

The present findings suggest that the behavioral expression and psychological significance of extraversion vary across sociocultural settings (Church, 2016). Tibetan communities are characterized by strong Buddhist traditions, close-knit interpersonal networks, and collectivistic social norms that emphasize social harmony, interpersonal obligation, and participation in community and religious activities (Markus and Kitayama, 1991; Samuel, 1993; Childs, 2004). Within this sociocultural context, greater sociability and interpersonal engagement may plausibly involve increased social responsibilities, stronger normative expectations, and more frequent participation in communal and religious practices (Triandis, 2001). Consequently, characteristics typically regarded as adaptive expressions of extraversion in individualistic societies may not necessarily confer the same psychological advantages in Tibetan communities (Church, 2016; Triandis, 2001). Rather than indicating that extraversion is inherently maladaptive, the present findings suggest that the psychological implications of extraverted behaviour may depend on the sociocultural context in which such behaviour is expressed. Accordingly, the observed association should be interpreted as evidence that the meaning and consequences of personality traits may not be culturally invariant. Similar perspectives have been proposed in cross-cultural personality research, suggesting that the behavioral manifestations and psychological implications of personality traits are shaped by culturally embedded social norms and values (Cheung et al., 2011; Triandis, 2001).

Nevertheless, this interpretation should be regarded as *post hoc* and interpreted cautiously. The present study was not designed to directly examine the cultural mechanisms underlying the observed association, and alternative explanations—including differences in personality measurement and construct representation across cultures—cannot be excluded (van de Vijver and Tanzer, 2004). Future research incorporating culturally validated personality measures together with direct assessments of social and religious experiences is needed to determine whether the positive association between extraversion and anxiety reflects genuine cultural variation in personality functioning or characteristics of the measurement instrument used in the present study (Hambleton et al., 2004).

### 4.4 Implications of the overlap between neuroticism and extraversion

An additional finding warranting careful consideration was the substantial overlap between neuroticism and extraversion observed in the present sample. These two personality dimensions were strongly positively correlated, and the exploratory factor analysis indicated considerable overlap between items theoretically assigned to each construct. Although regression diagnostics indicated that multicollinearity was not problematic, the observed overlap suggests that neuroticism and extraversion were less clearly differentiated than is typically reported in studies conducted in Western populations (Cheung et al., 2011; McCrae and Terracciano, 2005).

Accordingly, the standardized regression coefficients should be interpreted as representing the unique statistical contribution of each personality dimension after accounting for their shared variance, rather than as completely independent psychological effects. Several factors may account for the observed overlap, including cultural variation in personality organization (Markus and Kitayama, 1991), characteristics of the brief personality measure (Gosling et al., 2003), or limitations associated with the current Tibetan adaptation. Because no formal psychometric validation study of the Tibetan version has yet been published, these alternative explanations cannot be distinguished in the present study.

Future research should combine comprehensive personality inventories with rigorous cross-cultural psychometric validation to determine whether the overlap observed in the present study reflects genuine cultural variation in personality structure or characteristics of the measurement instrument.

### 4.5 Limited incremental contribution of social support and religious affiliation

Perceived social support and religious affiliation provided negligible additional explanatory value after personality traits had been taken into account. This finding should not be interpreted as evidence that these social-contextual factors are unimportant for mental health. Rather, it may reflect the limited sensitivity of the broad categorical indicators used in the present study, which were unlikely to capture the multidimensional nature of perceived social support and religiosity. Previous research has shown that both constructs encompass multiple structural and functional dimensions and that their associations with psychological outcomes depend substantially on measurement specificity (Cohen and Wills, 1985; Koenig, 2012). Consistent with this interpretation, neither perceived social support nor religious affiliation moderated the association between neuroticism and anxiety symptoms.

Within the present measurement framework, personality traits explained substantially more variance in anxiety symptoms than the broad indicators of perceived social support and religious affiliation included in the regression models. Future research should incorporate multidimensional and culturally appropriate assessments of perceived social support and religiosity to better evaluate their independent and potential interactive contributions to anxiety symptoms. Consequently, the present findings should not be interpreted as evidence that social support or religiosity are unimportant for anxiety, but rather that the relatively broad indicators available in the present study provided limited discriminatory value once personality traits had been taken into account.

### 4.6 Clinical and theoretical implications

The present findings should be interpreted in light of the measurement and cultural considerations discussed above. Nevertheless, several implications for research and practice emerge. First, personality traits demonstrated substantially greater explanatory value for anxiety symptoms than demographic, health-related, and broad social-contextual characteristics within the present measurement framework. These findings highlight the potential value of incorporating personality assessment into mental health screening, risk identification, and preventive intervention strategies. Individuals with elevated neuroticism may be particularly vulnerable to anxiety symptoms and may therefore benefit from interventions targeting emotion regulation, cognitive restructuring, stress management, and resilience enhancement (Kotov et al., 2010; Lahey, 2009).

The positive association between extraversion and anxiety further emphasizes the importance of culturally informed interpretations of personality traits. Personality constructs developed and validated primarily in Western populations may not necessarily have identical psychological meanings or implications across different sociocultural settings. The present findings suggest that the psychological correlates of extraversion may depend, at least in part, on local social norms, interpersonal expectations, and broader sociocultural contexts. More broadly, these findings reinforce the importance of evaluating personality constructs using culturally validated measurement approaches when extending personality research to understudied populations.

### 4.7 Limitations

Several limitations should be acknowledged when interpreting the present findings. First, the cross-sectional design precludes causal inference (Setia, 2016). Although neuroticism and extraversion were associated with anxiety symptoms, the temporal direction of these associations cannot be established. Longitudinal studies are needed to determine whether personality traits prospectively predict changes in anxiety symptoms.

Second, although translation, back-translation, and cultural adaptation procedures were undertaken, no formal psychometric validation study of the Tibetan adaptation of the Brief Big Five Personality Scale is currently available. In addition, both the neuroticism and extraversion subscales demonstrated modest internal consistency across Cronbach’s *α* and McDonald’s *ω* total estimates (Revelle and Condon, 2019). Modest reliability may nevertheless have attenuated the observed associations.

Third, neuroticism and extraversion were strongly correlated, and the exploratory factor analysis indicated substantial overlap between these constructs. Although multicollinearity diagnostics remained within acceptable limits, it remains unclear whether this pattern reflects genuine cultural variation in personality organization, characteristics of the brief personality measure, or limitations of the current Tibetan adaptation.

Fourth, mental health history, perceived social support, and religious affiliation were assessed using relatively broad self-reported indicators rather than validated multidimensional measures. Consequently, the non-significant findings for perceived social support and religious affiliation should be interpreted cautiously because limited measurement sensitivity may have reduced their explanatory value.

Finally, participants were recruited using convenience sampling, which may limit the generalizability of the findings and introduce potential response bias. Replication in larger and more representative Tibetan samples using culturally validated personality measures will be important for establishing the robustness and generalizability of the present findings (Bornstein et al., 2013). Because several of these limitations are interrelated, particularly the absence of formal psychometric validation and the use of brief contextual indicators, the present findings should be interpreted primarily as hypothesis-generating rather than definitive evidence regarding personality functioning in Tibetan populations.

## 5 Conclusion

In conclusion, the present study demonstrated that personality traits provided substantial incremental explanatory value for anxiety symptoms within the present measurement framework in a Tibetan sociocultural context. Neuroticism emerged as a robust positive correlate of anxiety, whereas extraversion was also positively associated with anxiety symptoms, a pattern that contrasts with findings commonly reported in Western populations. By contrast, the broad indicators of perceived social support and religious affiliation examined in the present study provided minimal additional explanatory value beyond personality traits. Collectively, these findings underscore the importance of considering both sociocultural context and measurement characteristics when interpreting personality–anxiety associations and support the use of culturally validated approaches to personality assessment and mental health research. Rather than assuming that personality–anxiety associations established in Western populations generalize universally, the present findings highlight the importance of evaluating personality constructs within their cultural and psychometric context before drawing conclusions regarding their psychological significance.

## Data Availability

The original contributions presented in the study are included in the article/Supplementary material, further inquiries can be directed to the corresponding author.
