# Supplementary material for: Personality traits and anxiety in a Tibetan cultural context: incremental contributions of neuroticism and extraversion
Source: Front Psychol. 2026 Aug 28;17:1906142. doi: 10.3389/fpsyg.2026.1906142 (PMC13562016; doi:10.3389/fpsyg.2026.1906142)
Supplement: Supplementary file 1 [file Supplementary_file_1.DOCX]

**eAppendix A.** Study Measures

**eAppendix B**. Reliability analyses

**eAppendix C.** Full Correlation Matrix

**eAppendix D.** Regression analyses, model diagnostics, and sensitivity analyses

**eAppendix E**. Exploratory moderation analyses

**eAppendix F.** Exploratory factor analysis

**eAppendix A.** Study Measures

| Table S1. Characteristics of study measures | | | | |
| --- | --- | --- | --- | --- |
| **Measure** | **Construct assessed** | **Items** | **Score range** | **Higher score indicates** |
| Brief Big Five Personality Scale (Neuroticism and Extraversion subscales) | Personality traits | 16(8 per subscale) | 1–6 per item | Higher levels of the corresponding trait |
| GAD-7 | Anxiety symptoms | 7 | 0–21 | Greater anxiety severity |
| Social support | Perceived social support | 1 | 1–3ᵃ | Greater social support |
| Religion | Religious affiliation | 1 | 1–2ᵇ | Not applicable |
| Note.ᵃ Social support was categorized as low, moderate, or high. ᵇ Religion was coded as 1 = religion and 2 = no religion. | | | | |

**eAppendix B**. Reliability analyses

| Table S2. Internal consistency of study measures | | |
| --- | --- | --- |
| **Variable** | **Cronbach's α** | **McDonald’s ω total** |
| Neuroticism | 0.67 | 0.67 |
| Extraversion | 0.69 | 0.70 |
| GAD-7 | 0.70 | — |
| Note. Cronbach’s α coefficients were calculated using the current sample (N = 583). McDonald’s ω total was estimated separately for each personality subscale using a one-factor minimum residual model based on Pearson correlations. Omega total was not calculated for the GAD-7. Values should be interpreted in light of the brevity of the personality subscales. | | |

**eAppendix C.** Full Correlation Matrix

| Table S3. Correlations among all study variables | | | | | | | | | | |
| --- | --- | --- | --- | --- | --- | --- | --- | --- | --- | --- |
| **Variable** | **1** | **2** | **3** | **4** | **5** | **6** | **7** | **8** | **9** | **10** |
| 1. Age | — |  |  |  |  |  |  |  |  |  |
| 2. Sex | .37*** | — |  |  |  |  |  |  |  |  |
| 3. Education | .43*** | .27*** | — |  |  |  |  |  |  |  |
| 4. Occupation | .41*** | .17*** | .52*** | — |  |  |  |  |  |  |
| 5. Income | .38*** | .30*** | .53*** | .37*** | — |  |  |  |  |  |
| 6. Mental health history | .03 | .14** | .04 | -.09* | .07 | — |  |  |  |  |
| 7. Social support | .29*** | .30*** | .30*** | .20*** | .39*** | .09* | — |  |  |  |
| 8. Religion | -.17*** | .12** | -.20*** | -.23*** | -.18*** | .37*** | -.07 | — |  |  |
| 9. Neuroticism | .14*** | .22*** | .08* | .01 | .26*** | .06 | .17*** | .04 | — |  |
| 10. Extraversion | .13** | .20*** | .07 | .01 | .22*** | .08* | .18*** | .05 | .78*** | — |
| 11. GAD-7 total score | .07 | .11* | .04 | .01 | .19*** | .06 | .10* | .01 | .60*** | .58*** |
| Note. N = 583. Pearson correlation coefficients are reported. Categorical variables were numerically coded for analysis. * p < .05 ** p < .01 *** p < .001 | | | | | | | | | | |

**eAppendix D.** Regression analyses, model diagnostics, and sensitivity analyses

| Table S4. Final hierarchical regression model predicting GAD-7 total score | | | | | | | |
| --- | --- | --- | --- | --- | --- | --- | --- |
| **Predictor** | **B** | **SE** | **β** | **t** | **p** | **Lower 95% CI** | **Upper 95% CI** |
| Age | -0.054 | 0.165 | -0.013 | -0.328 | .743 | -0.378 | 0.270 |
| Sex | -0.276 | 0.265 | -0.039 | -1.042 | .298 | -0.796 | 0.244 |
| Education | -0.093 | 0.166 | -0.024 | -0.560 | .576 | -0.419 | 0.233 |
| Occupation | 0.003 | 0.120 | .001 | 0.023 | .981 | -0.234 | 0.239 |
| Income | 0.334 | 0.209 | .068 | 1.600 | .110 | -0.076 | 0.743 |
| Mental health history | 0.256 | 0.326 | .028 | 0.787 | .432 | -0.384 | 0.896 |
| Neuroticism | 1.712 | 0.246 | .368 | 6.965 | <.001 | 1.229 | 2.195 |
| Extraversion | 1.353 | 0.245 | .289 | 5.533 | <.001 | 0.873 | 1.834 |
| Social support | -0.141 | 0.258 | -0.020 | -0.546 | 0.585 | -0.647 | 0.366 |
| Religion | -0.186 | 0.340 | -0.020 | -0.546 | 0.585 | -0.854 | 0.482 |
| Note. Results correspond to the final hierarchical regression model (Model 3). B = unstandardized coefficient; SE = standard error; β = standardized coefficient. The final model explained 39.4% of the variance in GAD-7 scores. | | | | | | | |

Figure S1

Normal P–P plot of standardized residuals

Figure S2

Scatterplot of standardized residuals versus standardized predicted values

| Table S6. Sensitivity analysis using dummy-coded sex and occupation | | | | | | | |
| --- | --- | --- | --- | --- | --- | --- | --- |
| Panel A. Model fit statistics | | | | | | | |
| **Model** | **R²** | **Adjusted R²** | **ΔR²** | **ΔF** | **df1** | **df2** | **p for ΔF** |
| Model 1 | .057 | .039 | .057 | 3.15 | 11 | 571 | <.001 |
| Model 2 | .402 | .389 | .345 | 164.35 | 2 | 569 | <.001 |
| Model 3 | .403 | .387 | 0 | 0.11 | 2 | 567 | 0.895 |

| Panel B. Final model coefficients | | | | | | | | |
| --- | --- | --- | --- | --- | --- | --- | --- | --- |
| **Predictor** | **B** | **SE** | **β** | **t** | **p** | **Lower 95% CI** | **Upper 95% CI** | **VIF** |
| Age | -0.095 | 0.168 | −.023 | -0.566 | 0.571 | -0.426 | 0.235 | 1.551 |
| Education | -0.008 | 0.172 | −.002 | -0.044 | 0.965 | -0.345 | 0.33 | 1.914 |
| Income | 0.348 | 0.209 | 0.07 | 1.663 | 0.097 | -0.063 | 0.758 | 1.697 |
| Mental health history | 0.297 | 0.325 | 0.033 | 0.913 | 0.362 | -0.342 | 0.936 | 1.212 |
| Female | -0.057 | 0.341 | −.006 | -0.166 | 0.868 | -0.727 | 0.614 | 1.335 |
| Prefer not to disclose sex | -0.627 | 0.605 | −.039 | -1.036 | 0.301 | -1.817 | 0.562 | 1.359 |
| Manual/service worker | 0.009 | 0.484 | 0.001 | 0.019 | 0.985 | -0.941 | 0.96 | 1.629 |
| Administrative/professional/technical staff | -0.127 | 0.496 | −.011 | -0.255 | 0.798 | -1.101 | 0.847 | 1.736 |
| Self-employed/freelancer | -0.326 | 0.626 | −.021 | -0.521 | 0.603 | -1.556 | 0.904 | 1.581 |
| Student | -1.855 | 0.874 | −.080 | -2.123 | 0.034 | -3.57 | -0.139 | 1.343 |
| Unemployed/retired | 0.88 | 0.696 | 0.047 | 1.264 | 0.207 | -0.487 | 2.246 | 1.336 |
| Neuroticism | 1.762 | 0.246 | 0.379 | 7.161 | <.001 | 1.279 | 2.246 | 2.663 |
| Extraversion | 1.334 | 0.245 | 0.285 | 5.451 | <.001 | 0.853 | 1.814 | 2.598 |
| Social support | -0.057 | 0.263 | −.008 | -0.219 | 0.827 | -0.573 | 0.458 | 1.325 |
| Religion | -0.146 | 0.344 | −.016 | -0.426 | 0.671 | -0.821 | 0.529 | 1.346 |
| Note. Sensitivity analyses were conducted by replacing the original numerically coded sex and occupation variables with dummy-coded variables. Male participants served as the reference group for sex, and agricultural/pastoral workers served as the reference group for occupation. Model 1 included demographic and clinical covariates; Model 2 additionally included neuroticism and extraversion; Model 3 additionally included social support and religion. B = unstandardized coefficient; SE = standard error; β = standardized coefficient; VIF = variance inflation factor. | | | | | | | | |

**eAppendix E**. Exploratory moderation analyses

| Table S5. Exploratory moderation analyses | | | | | | | | | |
| --- | --- | --- | --- | --- | --- | --- | --- | --- | --- |
| **Moderator** | **Interaction term** | **B** | **SE** | **β** | **t** | **p** | **ΔR²** | **F change** | **p for ΔR²** |
| Social support | Neuroticism × Social support | -0.028 | 0.274 | -0.009 | -0.104 | .918 | .000 | 0.011 | .918 |
| Religion | Neuroticism × Religion | 0.494 | 0.317 | .162 | 1.557 | 0.120 | .003 | 2.425 | .120 |
| Note. Neuroticism was mean-centered prior to the creation of interaction terms. Each interaction term was entered separately after the covariates, personality traits, social support, and religion. ΔR² refers to the change in explained variance after adding the interaction term. | | | | | | | | | |

**eAppendix F.** Exploratory factor analysis

F1. Factorability

The Kaiser–Meyer–Olkin (KMO) measure was 0.832, and Bartlett’s test of sphericity was significant (χ² = 2083.37, p < .001), indicating that the data were suitable for factor analysis.

F2. Extraction method

Exploratory factor analysis (EFA) was conducted using principal axis factoring with Promax rotation. Factor retention was initially evaluated using the Kaiser criterion (eigenvalues > 1) together with visual inspection of the scree plot.

F3. Factor retention

The Kaiser criterion initially suggested a four-factor solution accounting for 52.33% of the total variance. However, inspection of the scree plot indicated a clear inflection after the second factor, and the four-factor solution had limited conceptual interpretability because its fourth factor was defined by only two items (Table S9). Accordingly, a theoretically informed two-factor solution was retained for interpretation. The two retained factors explained 37.53% of the total variance (Table S7).

| Table S7. Total variance explained | | | |
| --- | --- | --- | --- |
| **Factor** | **Eigenvalue** | **% of Variance** | **Cumulative %** |
| 1 | 4.464 | 27.9 | 27.9 |
| 2 | 1.541 | 9.63 | 37.53 |

F4. Pattern matrix

| Table S8. Pattern matrix of exploratory factor analysis | | |
| --- | --- | --- |
| **Item** | **Factor 1 (Neuroticism-related)** | **Factor 2 (Extraversion-related)** |
| N2 | **0.755** |  |
| N4 | **0.738** |  |
| N6 | **0.564** |  |
| E1 | **0.531** |  |
| N7 |  | **0.78** |
| N3 |  | **0.47** |
| N5 |  | **0.459** |
| E2 |  | **0.432** |
| N1 |  | **0.422** |
| E7 |  | **0.408** |
| Note. Factor loadings < .30 were suppressed. Items with no factor loading ≥ .30 are not shown. Primary loadings are shown in bold. | | |

Initial extraction based on the Kaiser criterion (eigenvalues > 1) yielded a four-factor solution. Inspection of the rotated pattern matrix showed that neuroticism- and extraversion-related items were distributed across multiple factors, and the fourth factor was defined by only two items. The initial four-factor pattern matrix is presented in Table S9.

| Table S9. Pattern matrix for the initial four-factor solution | | | | |
| --- | --- | --- | --- | --- |
| **Item** | **Factor 1** | **Factor 2** | **Factor 3** | **Factor 4** |
| N4 | 0.654 |  |  |  |
| N2 | 0.648 |  |  |  |
| N6 | 0.542 |  |  |  |
| E1 | 0.503 |  |  |  |
| E4 | 0.380 |  |  |  |
| E6 |  | 0.608 |  |  |
| N7 |  | 0.595 |  |  |
| E2 |  | 0.409 |  |  |
| E8 |  | 0.395 |  |  |
| N8 |  | 0.375 |  |  |
| E3 |  |  | 0.621 |  |
| N5 |  |  | 0.612 |  |
| E5 |  |  | 0.370 |  |
| N1 |  |  |  | 0.748 |
| N3 |  |  |  | 0.356 |
| Note. The initial extraction retained four factors according to the Kaiser criterion (eigenvalues > 1). Extraction method: Principal axis factoring. Rotation method: Promax with Kaiser normalization. Factor loadings < .30 are suppressed. | | | | |

F5. Factor correlations

The two factors were moderately correlated (r = 0.536), indicating a substantial association between the extracted dimensions.

| Table S10. Factor correlation matrix | | |
| --- | --- | --- |
| **Factor** | **1** | **2** |
| 1 | 1.00 | .536 |
| 2 | .536 | 1.00 |

F6. Interpretation

The retained two-factor solution suggested substantial overlap between neuroticism and extraversion, with several items loading on factors that did not fully correspond to their theoretical assignments. This pattern may reflect cultural variation in personality organization, characteristics of the brief personality measure, or limitations of the current Tibetan adaptation.
